# A multiomics analysis of S100 protein family in breast cancer

**DOI:** 10.18632/oncotarget.25561

**Published:** 2018-06-26

**Authors:** Patrizia Cancemi, Miriam Buttacavoli, Gianluca Di Cara, Nadia Ninfa Albanese, Serena Bivona, Ida Pucci-Minafra, Salvatore Feo

**Affiliations:** ^1^ Department of Biological Chemical and Pharmaceutical Sciences and Technologies (STEBICEF), University of Palermo, Palermo, Italy; ^2^ Center of Experimental Oncobiology (C.OB.S.), La Maddalena Hospital III Level Oncological Dept., Palermo, Italy; ^3^ Advanced Technologies Network Center (ATeN), University of Palermo, Palermo, Italy; ^4^ Institute of Biomedicine and Molecular Immunology, CNR, Palermo, Italy

**Keywords:** S100 proteins, breast cancer, expression analysis, proteomics, pathway analysis

## Abstract

The S100 gene family is the largest subfamily of calcium binding proteins of EF-hand type, expressed in tissue and cell-specific manner, acting both as intracellular regulators and extracellular mediators. There is a growing interest in the S100 proteins and their relationships with different cancers because of their involvement in a variety of biological events closely related to tumorigenesis and cancer progression. However, the collective role and the possible coordination of this group of proteins, as well as the functional implications of their expression in breast cancer (BC) is still poorly known. We previously reported a large-scale proteomic investigation performed on BC patients for the screening of multiple forms of S100 proteins. Present study was aimed to assess the functional correlation between protein and gene expression patterns and the prognostic values of the S100 family members in BC. By using data mining, we showed that S100 members were collectively deregulated in BC, and their elevated expression levels were correlated with shorter survival and more aggressive phenotypes of BC (basal like, HER2 enriched, ER-negative and high grading). Moreover a multi-omics functional network analysis highlighted the regulatory effects of S100 members on several cellular pathways associated with cancer and cancer progression, expecially immune response and inflammation. Interestingly, for the first time, a pathway analysis was successfully applied on different omics data (transcriptomics and proteomics) revealing a good convergence between pathways affected by S100 in BC. Our data confirm S100 members as a promising panel of biomarkers for BC prognosis.

## INTRODUCTION

Breast cancer is the most diagnosed and potentially aggressive form of cancer in women [1]. Although genetic alterations in proto-oncogenes, tumor suppressor genes, cell cycle regulators and cell growth factors have been implicated in the process of carcinogenesis, the progression toward full malignancy is extremely complex at molecular level [2]. Different protein factors can be over/under expressed simultaneously and activate/deactivate different cell functions. Breast cancer is a multifaceted disease of distinct biological subtypes with different clinical, pathological, molecular features and proteomic differences [3–9]. The molecular classification of breast cancer, based on the expression of estrogen/progesterone receptor (ER/PR) and epidermal growth factor receptor 2 (HER2), provides different prognostic/predictive implications and therapeutic informations. Despite these advances, breast cancer remains one of the most enigmatic and poorly predictable cancer in its evolution due to the elevated biological heterogeneity consistent with observed varied responses to therapies across patients [6]. Thus novel biomarkers useful in clinical setting and/or for breast cancer management are coming up to explore.

One class of protein with emerging roles in breast cancer is the S100 family, a multigenic family of Ca^2+^ binding proteins of the EF-hand type, comprising at least 20 members [10]. The majority of them (S100A1-S100A16) are coded by genes that clustered at chromosome locus 1q21 (known as epidermal differentiation complex), while the others (S100B, S100G, S100P and S100Z) are located in other chromosome loci, 21q22, Xp22, 4p16 and 5q14, respectively [11]. It is well documented that S100 proteins have a broad range of intracellular and extracellular functions, and are implicated in multiple biological functions, including cell division, motility, secretion, protein synthesis, and membrane permeability [12]. In addition, recent studies have reported the association between S100 family members and breast cancer development and progression [13–15]. Despite the promising potential of the S100 family as a biomarker panel, there are few studies that have addressed the family-wide expression of S100 protein isoforms in clinical samples [16–20]. We recently reported a large proteomic screening for S100 protein expression on breast cancer patients [21]. The results showed that some protein members are ubiquitously expressed in almost all patients, while others appeared more sporadic within the same group, and most of the detected S100 members appeared reciprocally correlated. More interestingly, patients which developed distant metastases showed a general tendency of higher S100 protein expression, compared to the disease-free group.

However, the mode of action of S100 proteins in breast cancer as well as the functional implications of alteration of gene expression levels remain to be elucidated.

Here, we performed a deep *in silico* analysis on the transcriptional profiles of 20 S100 family members (S100A1-S100A16, S100B, S100G, S100P and S100Z) between cancer and normal tissues. Moreover, S100 gene expression levels were correlated to the clinic-pathological features (Molecular subtypes, ER status, Grading) and survival data, evaluated as Overall Survival (OS), Distant Metastasis Free Survival (DMFS), and Relapse Free Survival (RFS). Finally, by using three cross platforms (GOBO, ONCOMINE and STRING databases) and our previous proteomic data, we explored the S100-regulated networks and pathways. Our results revealed that S100 genes were de-regulated in BC patients compared with normal tissues and collectively were over-expressed in HER2 enriched and Basal-like subtypes. In survival analysis, high transcriptional levels of S100 genes were associated with worse prognosis, probably because S100 expression affects immune response and inflammatory pathways. This study represents the first multi-omics attempt capable of revealing an integrated view of biological mechanisms regulated by S100 protein family and meaningful the important involvement of S100 family in breast cancer progression.

## RESULTS

### Gene expression analysis of S100 family members between normal and cancer tissues

**Figure 1:**
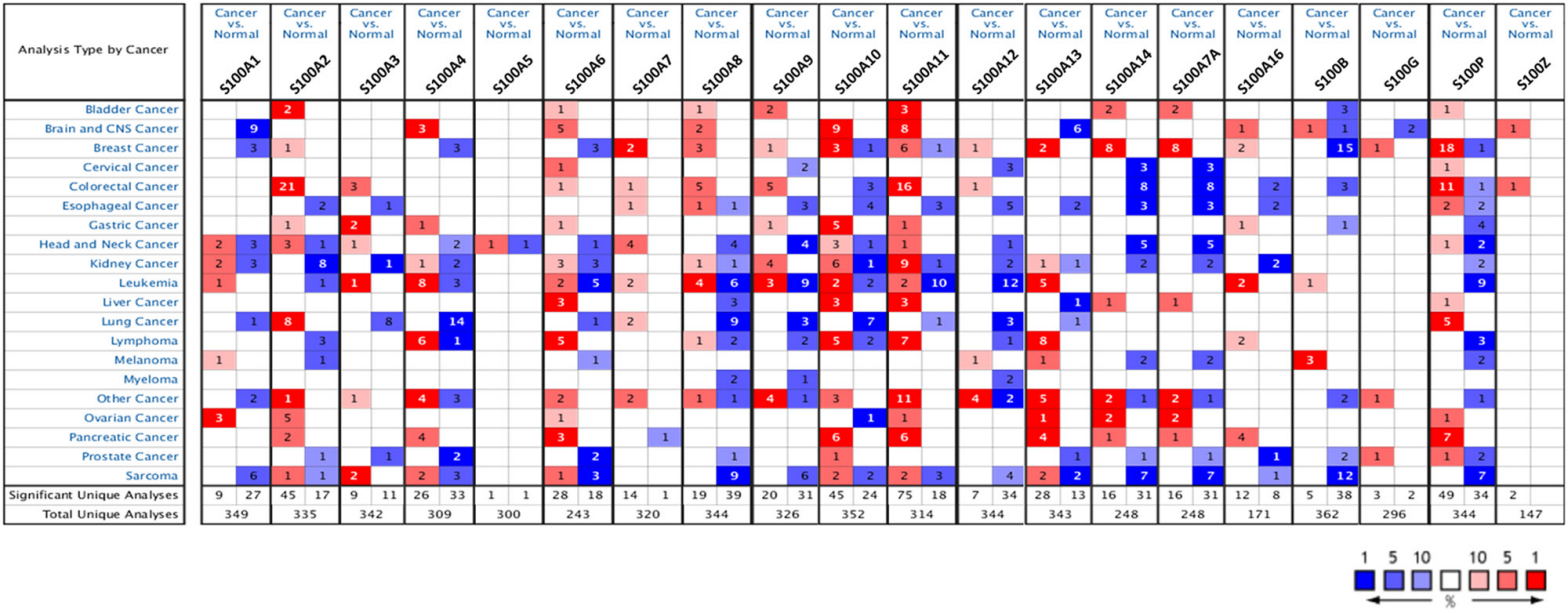
Gene expression analysis of S100 family members between normal and cancer tissues The mRNA expression differences between tumors and normal tissues were analyzed for each S100 gene in ONCOMINE database using the following thresholds: *p*-value: 1E-4; fold change: 2; gene rank: top 10%; data type: mRNA; sample type: clinical specimens. The number in the colored box represents the number of analyses meeting these thresholds. The color depth was determined by the gene rank. The red boxes indicate that the mRNA levels of target genes are higher in tumor tissues than in normal tissues, while blue boxes indicate that the mRNA levels of target genes are lower in tumor tissues than in normal tissues.

The transcription levels of the S100 family members between tumor and normal tissues in multiple cancers was compared by using ONCOMINE database [22]. As shown in Figure 1, the database performed a total of 6037 unique analyses for all the S100 genes across a wide variety of datasets in different cancer types and 840 showed a significant statistical difference for mRNA expression. In particular, S100 family members were found up-regulated in 429 analyses, and down-regulated in 411 cancer versus normal analyses. These results suggest that S100 family members might play important roles during carcinogenesis in different cancer types, acting both as oncogenes or suppressor genes. In particular, for breast cancer, ONCOMINE analysis revealed that collectively S100 mRNA expression was higher in tumoral than normal samples (55 analyses with up-regulation versus 27 analyses with down-regulation) and only S100A3 and S100A5 did not show differences between the analyzed dataset.

### Genomic alterations of S100 family members in breast cancer

**Figure 2:**
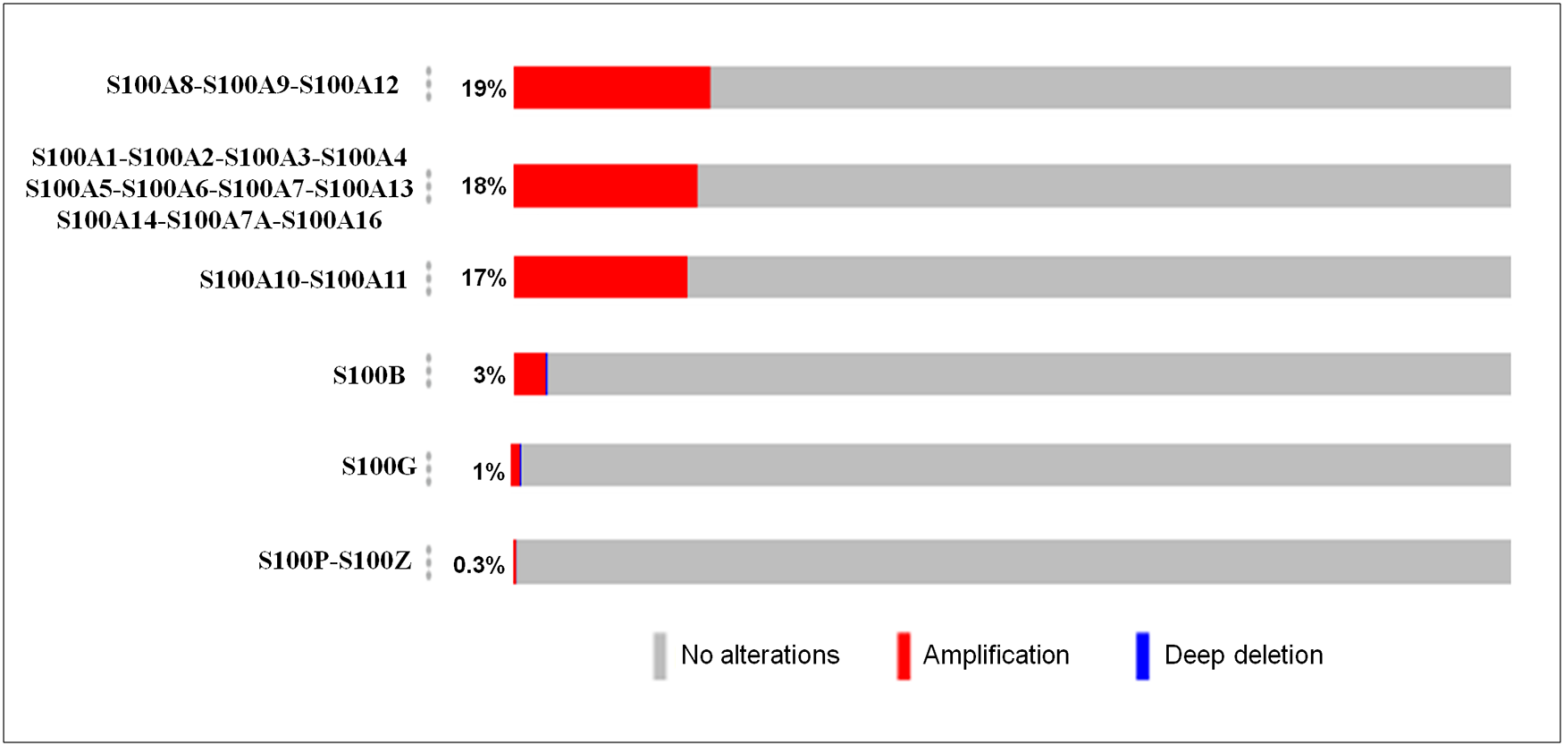
Genetic alterations of S100 genes in breast cancer The percentage of alterations in S100 genes was extracted by using the OncoPrint tool of cBioportal containing sequencing data of 2509 patients. Red and blue represent amplification and deep deletion, respectively.

The OncoPrint tool of cBioPortal database [23] was used to query for alterations in S100 genes in breast cancer. As shown in Figure 2, the percentage of alterations spans from 0.3% to 19% for individual genes. In particular, the predominant pattern of amplification occurred in 19%, 18% and 17% of the S100 genes clustering into 1p chromosome, while a low percentage of alterations that include both gene amplifications and gene deletions were recorded for the other S100 genes.

### S100s gene expression is associated with clinical parameters

**Figure 3:**
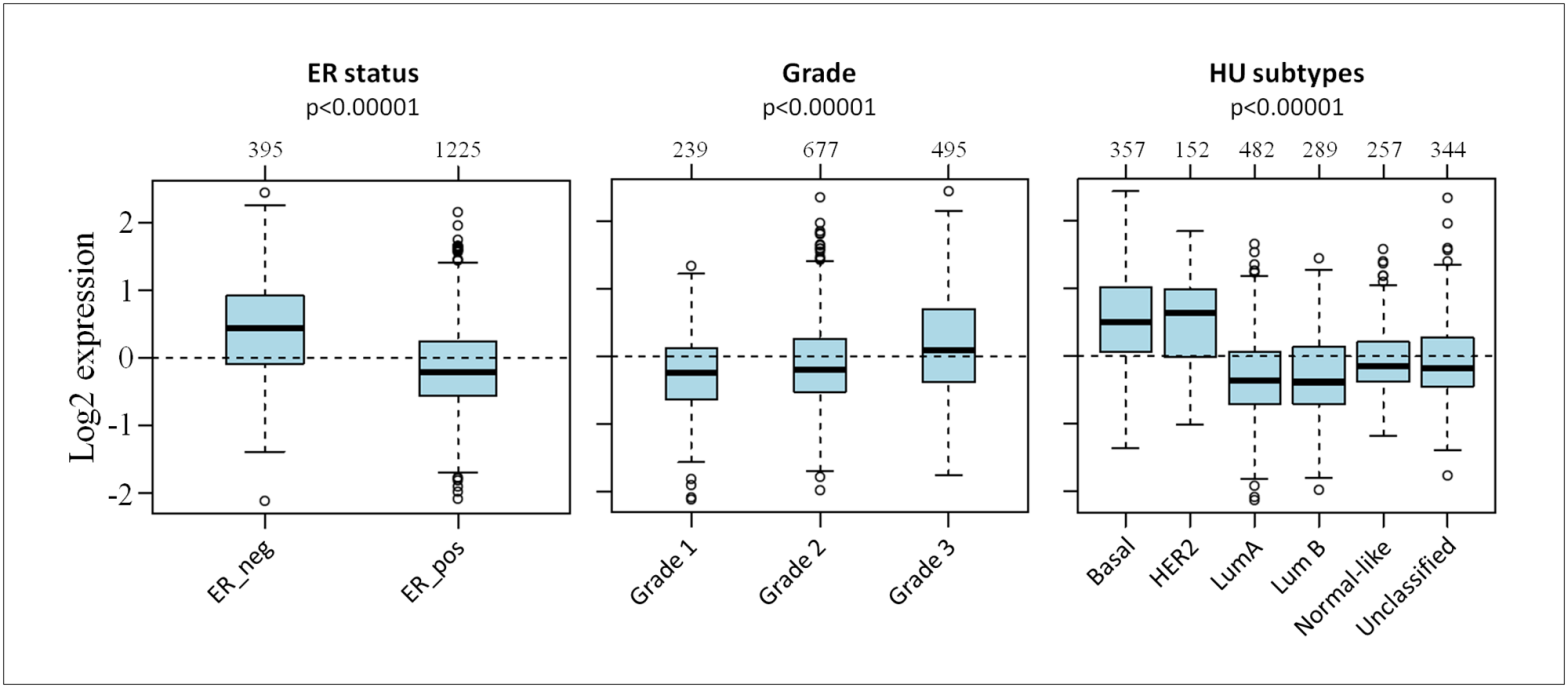
Association between S100s gene expression and clinical parameters Box Plot of the mRNA expression of S100 family members. Patients were stratified according ER status, tumor grading and HU subtypes applying the gene Set Analysis (GSA) of GOBO database.

We then used GOBO database, containing 1881 patient’s data [23], to investigate the correlation between S100s gene expression and clinical parameters, including ER expression, tumor grade and molecular subtypes. We found significantly higher S100 expression levels (Figure 3) in ER negative tumors, in higher grade tumors and in basal-like and HER2 tumors (p<0.0001 according to one-way ANOVA), while lower S100 expression levels were found in Luminal A and Luminal B tumors.

### Prognostic significance of S100 family members

We also investigated the prognostic value of S100 family members in breast cancer using survival data, evaluated as Overall Survival (OS), Distant Metastasis Free Survival (DMFS) and Relapse Free Survival (RFS), of 5143 breast patients from Kaplan-Meier plotter database [24]. Firstly, the survival analysis was performed for each S100 gene, using a single Affimetrix ID probe. Results shown in Table 1, clearly indicate the association between the higher S100s expression with a worse prognosis (highlighted in red) or better prognosis (highlighted in black), pointed about the specific roles of single members as oncogenes or tumor suppressor genes. No association with survival data was recorded for S100A2, S100A3, S100A13, S100A14, S100A7A and S100B.

**Table 1:** Correlation of S100s with survival outcomes in breast cancer patients

Gene ID	Affimetrix ID	Survival outcome	Number of cases	Cut-off	Expression range of the probe	HR	LogrankP
**S100A1**	205334-at	RFS	3955	262	4-14584	0.87	**0.016**
		DMFS	1746	274	4-14584	0.92	0.38
		OS	1402	233	4-10656	0.74	**0.006**
**S100A2**	204268-at	RFS	3955	269	3-72629	0.95	0.31
		DMFS	1746	284	7-33668	0.97	0.74
		OS	1402	308	6-72629	0.98	0.85
**S100A3**	206027-at	RFS	3955	68	3-7734	0.94	0.26
		DMFS	1746	68	4-1799	1.07	0.48
		OS	1402	80	3-1799	0.95	0.64
**S100A4**	203186-s-at	RFS	3955	2626	37-33152	1.12	**0.044**
		DMFS	1746	2597	122-27019	1.1	0.33
		OS	1402	2840	122-27019	0.97	0.77
**S100A5**	207763-at	RFS	3955	14	1-314	0.87	**0.012**
		DMFS	1746	14	2-314	0.97	0.76
		OS	1402	16	2-291	1	0.98
**S100A6**	217728-at	RFS	3955	5323	418-52048	1.07	0.2
		DMFS	1746	5293	739-49544	1.02	0.81
		OS	1402	5824	419-50535	0.81	**0.049**
**S100A7**	205916-at	RFS	3955	39	1-55811	1.17	**0.0038**
		DMFS	1746	46	2-52684	1.26	**0.0019**
		OS	1402	79	2-55811	1.23	0.062
**S100A8**	202917-s-at	RFS	3955	514	3-128800	1.45	**2.60E-11**
		DMFS	1746	545	2-55811	1.38	**0.0012**
		OS	1402	700	3-128800	1.4	**0.0021**
**S100A9**	203535-at	RFS	3955	341	5-94557	1.41	**5.50E-10**
		DMFS	1746	331	5-43408	1.41	**0.00047**
		OS	1402	430	13-94557	1.38	**0.0031**
**S100A10**	200872-at	RFS	3955	6750	522-38772	1.35	**8.00E-08**
		DMFS	1746	6626	522-28029	1.43	**0.00031**
		OS	1402	6383	522-38778	1.26	**0.037**
**S100A11**	200660-at	RFS	3955	8844	162-57630	1.45	**3.10E-11**
		DMFS	1746	8468	302-57630	1.41	**0.00052**
		OS	1402	8844	241-33276	1.28	**0.022**
**S100A12**	205863-at	RFS	3955	83	1-3261	0.78	**1.20E-05**
		DMFS	1746	83	3-1850	1.2	0.068
		OS	1402	93	2-2811	1.15	0.2
**S100A13**	202598-at	RFS	3955	2672	286-20048	0.94	0.25
		DMFS	1746	2743	475-20048	0.83	0.059
		OS	1402	2607	475-15774	0.89	0.27
**S100A14**	218677-at	RFS	3955	1611	17-13313	1.05	0.37
		DMFS	1746	1720	76-12605	1.01	0.89
		OS	1402	1556	54-12605	0.99	0.9
**S100A7A**	232170-at	RFS	3955	21	11-11406	0.91	0.21
		DMFS	1746	31	1-11406	1.31	0.1
		OS	1402	28	1-11406	0.89	0.45
**S100A16**	227998-at	RFS	3955	4529	239-18268	1.23	**0.008**
		DMFS	1746	4829	683-16912	1.15	0.4
		OS	1402	4102	290-14364	1.27	0.13
**S100B**	209686-at	RFS	3955	26	1-16122	1.03	0.59
		DMFS	1746	28	1-3366	0.89	0.22
		OS	1402	26	1-8014	0.87	0.21
**S100G**	207885-at	RFS	3955	15	1-18316	0.77	**4.20E-06**
		DMFS	1746	15	1-13476	0.92	0.42
		OS	1402	14	1-12435	0.93	0.48
**S100P**	204351-at	RFS	3955	1117	3-46947	1.5	**2.60E-13**
		DMFS	1746	1113	6-29328	1.36	**0.0019**
		OS	1402	1113	5-44788	1.63	**7.20E-16**
**S100Z**	1554876-a-at-at	RFS	3955	25	1-398	0.72	**4.80E-05**
		DMFS	1746	23	1-139	0.7	**0.03**
		OS	1402	22	1-139	0.88	0.44

The prognostic value of individual S100 members evaluated in KM plotter database. For each S100 (identified with an Affimetrix ID) survival outcome was evaluated as Relapse Free Survival (RSF), Distant Metastasis Free Survival (DMFS) and Overall Survival (OS). Patients were splitted by median. Significant associations with prognosis are underlined in bold.

**Figure 4:**
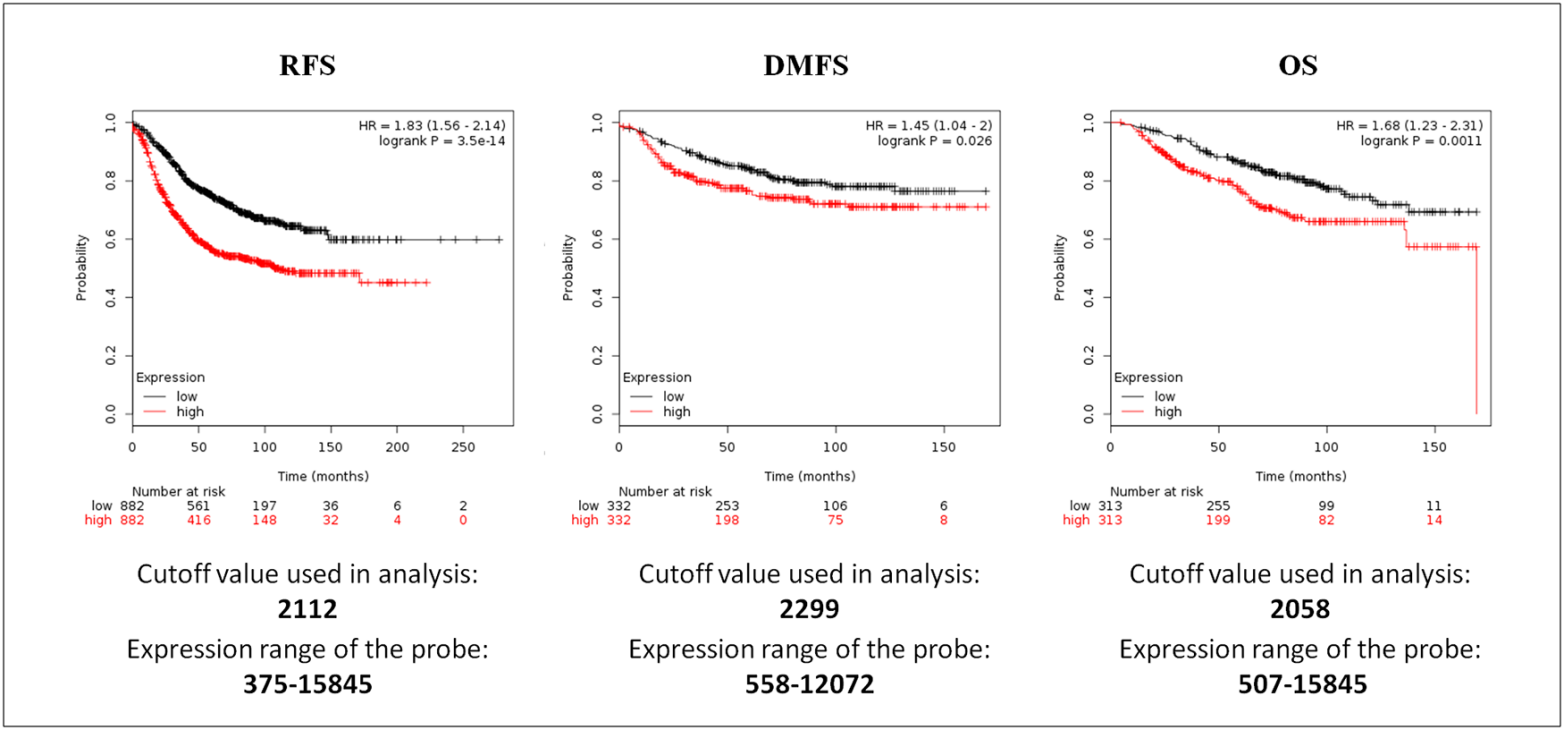
Survival analyses of S100 family members in breast cancer obtained from the Kaplan-Meier Plotter database Survival was evaluated as RFS (relapse free survival), DMFS (distant metastasis free survival) and OS (overall survival). Patients were splitted into two groups by using the best cut-off of probe expression.

Interestingly, when the survival analysis was performed including all the S100s members, higher expression levels of S100s members, were significantly correlated with a shorter RSF, with HR = 1.83 (1.56–2.14), p = 3.5E^-14^, DMFS with HR = 1.45 (1.04–2), p = 0.026 and OS with HR = 1.68 (1.23–2.31), p = 0.0011 (Figure 4). These results revealed that collectively S100 family members have a greater prognostic value than the individual genes.

**Figure 5:**
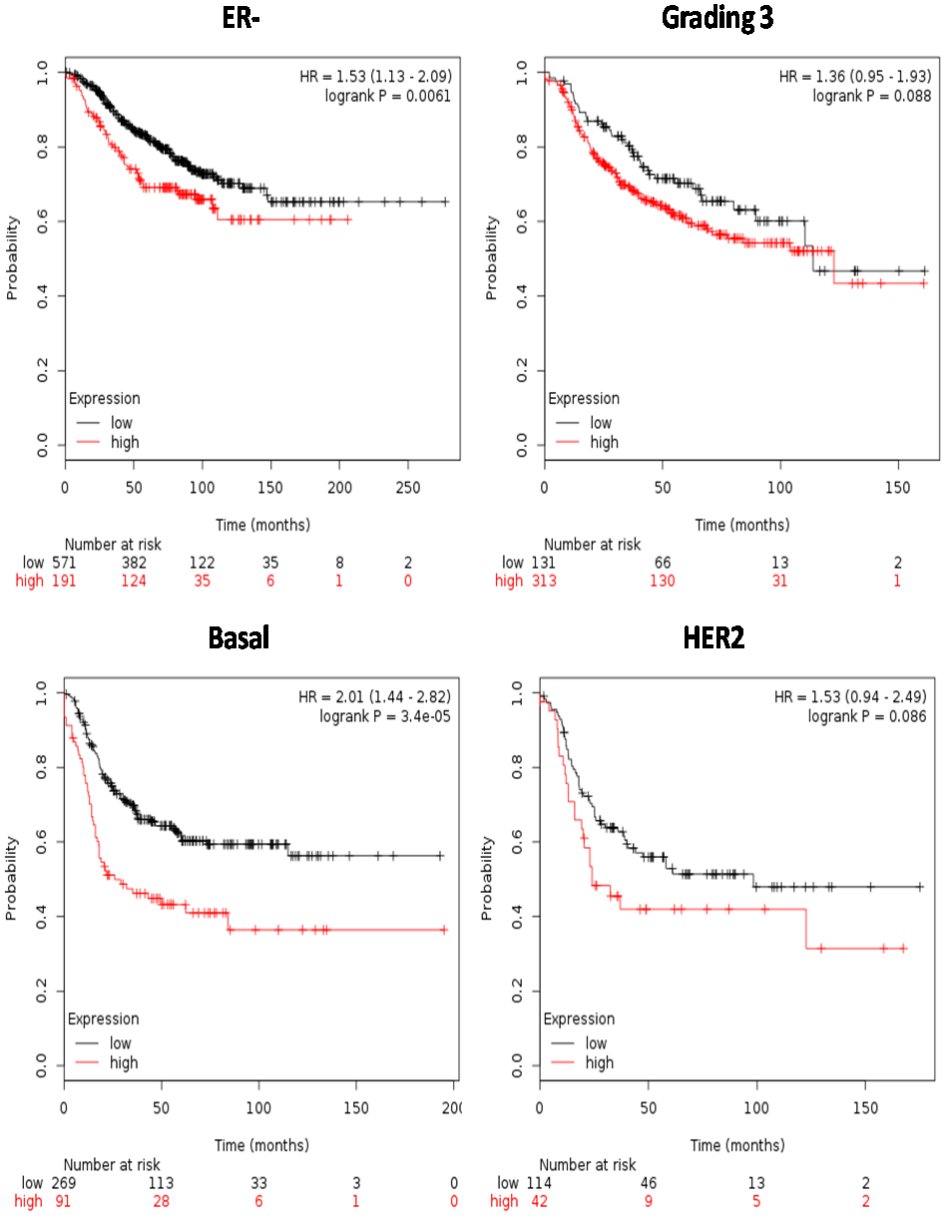
Survival analyses of S100 family members in breast cancer subgroups from the Kaplan-Meier Plotter database Survival was evaluated as RFS (relapse free survival). Patients were splitted into two groups by using the best cut-off of probe expression.

Survival analyses were carried out on the subgroup of patients that in GOBO analysis showed significant S100 up-regulation. Intriguingly, up-regulated S100s were all significantly associated with worse RFS in the ER- and basal-like tumors (Figure 5), but not in HER2-enriched group or in high grading, where the no statistical difference were recorded (p>0.05).

### Interaction network and pathway analysis of S100 family members

**Figure 6:**
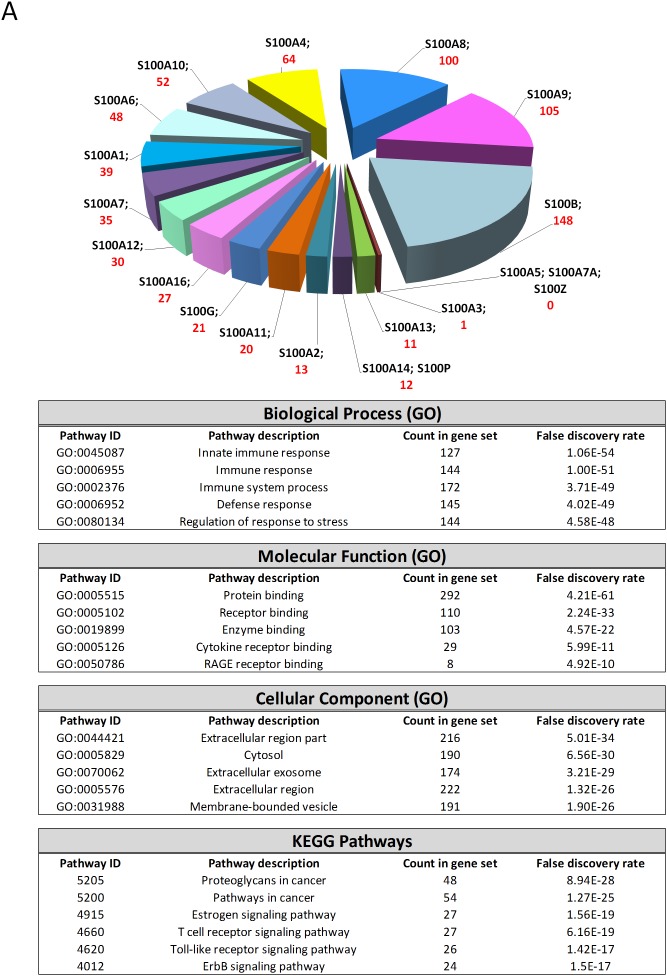

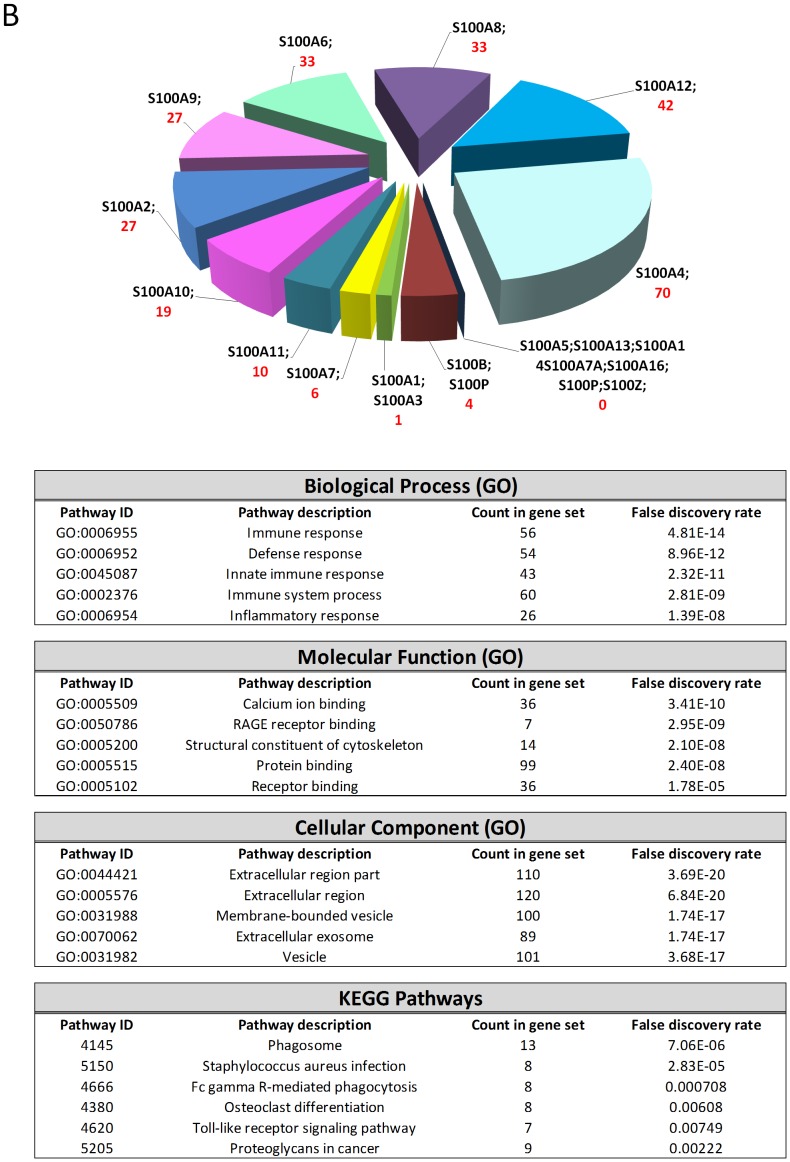

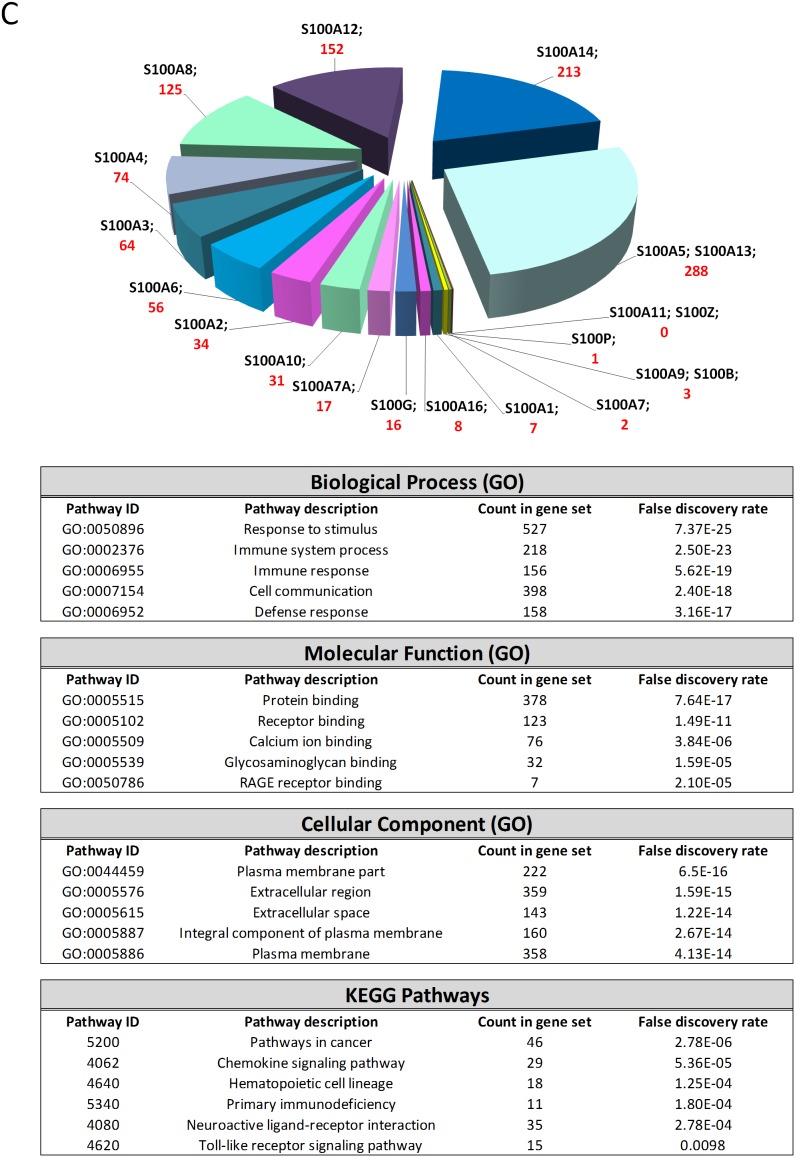
S100-associated genes analyzed with STRING (A), GOBO (B) and ONCOMINE (C) databases Graphs represent the number of associated genes (STRING) and co-expressed genes (GOBO and ONCOMINE) for each S100. The tables represent the 5 top significantly biological processes, molecular functions, cellular components and key pathways evaluated by using the String Analysis tool.

We then analyzed the possible interactions between S100 family members with other genes via computational analysis by using three cross-platforms (STRING, GOBO and ONCOMINE), in order to reveal networks and pathways able to predict the underlying molecular mechanisms of S100-mediated roles in breast cancer. The predicted associations and the co-expressed genes for each S100 were queried to STRING, GOBO and ONCOMINE database, respectively, by using a combined score of ≥ 0.4. The associations in STRING include direct (physical) interactions, as well as indirect (functional) interactions, as long as both are specific and biologically meaningful. The co-expressed genes in GOBO and in ONCOMINE databases are calculated by Pearson correlation method. For each S100, the databases returned the predicted functional associations and the co-expressed genes, as reported in the pie chart of Figure 6. The lists of the S100-interactors from STRING, and the co-expressed genes from GOBO and ONCOMINE (Supplementary Tables 1-3) (containing 547, 289 and 1261 unique proteins) were used to found functional enrichments in the S100-networks and clusterized through GO classification and KEGG pathways by using the String Analysis tool (Figure 6). Although each S100 was significantly associated with different genes in different databases, they are implicated in the same biological functions: infact, collectively, S100 affect inflammatory and immune response pathways, probably acting extracellularly, through the Toll-like and RAGE signaling.

**Figure 7:**
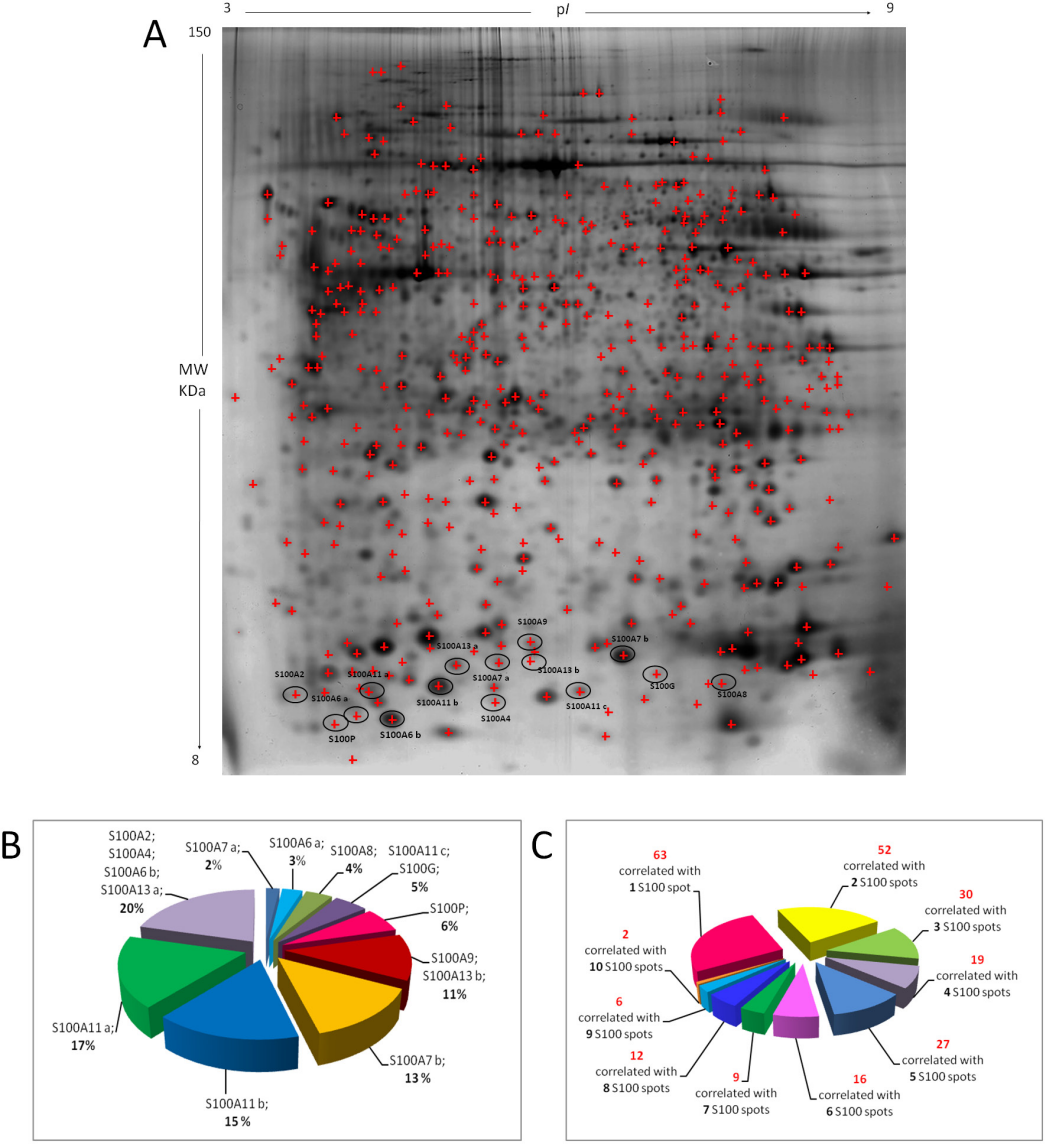

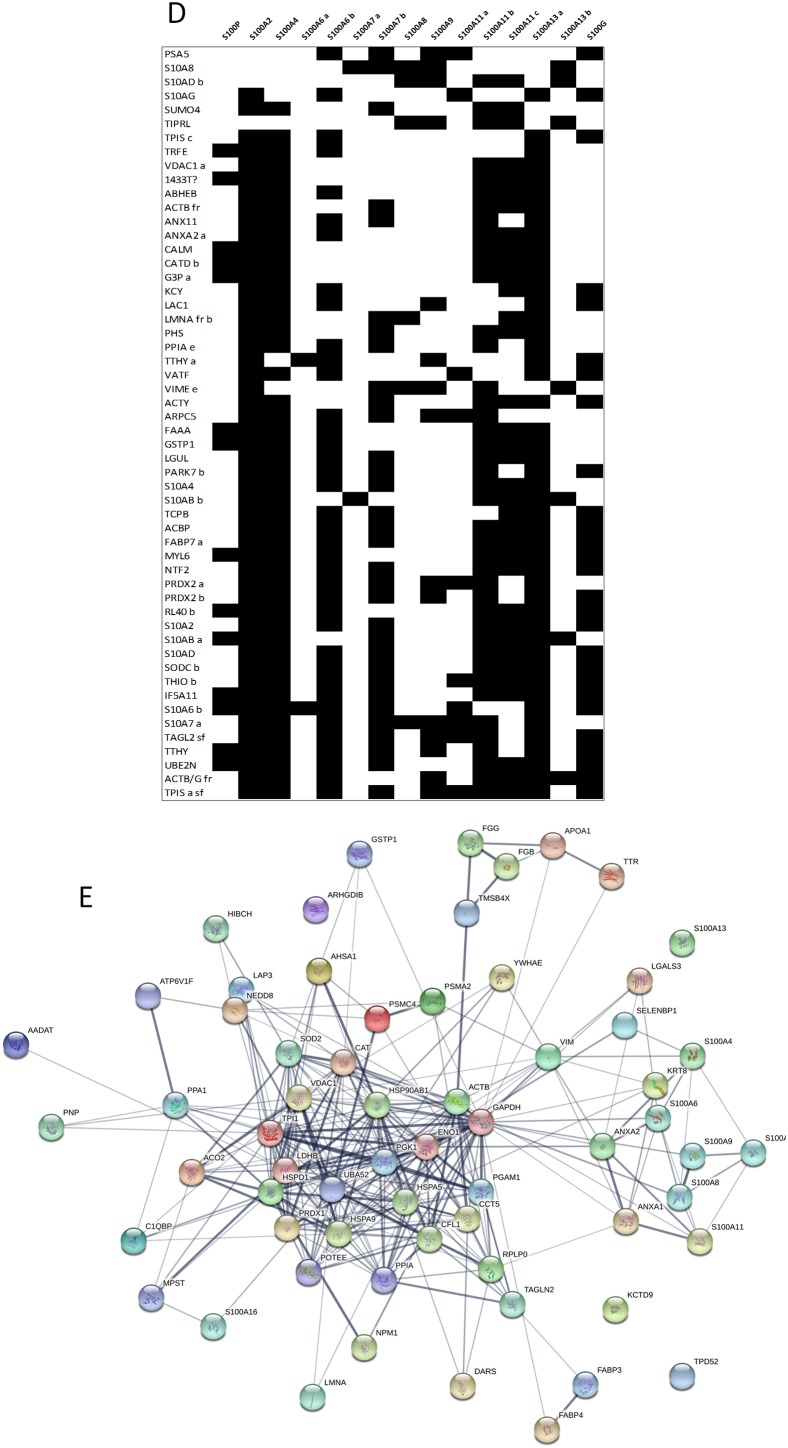

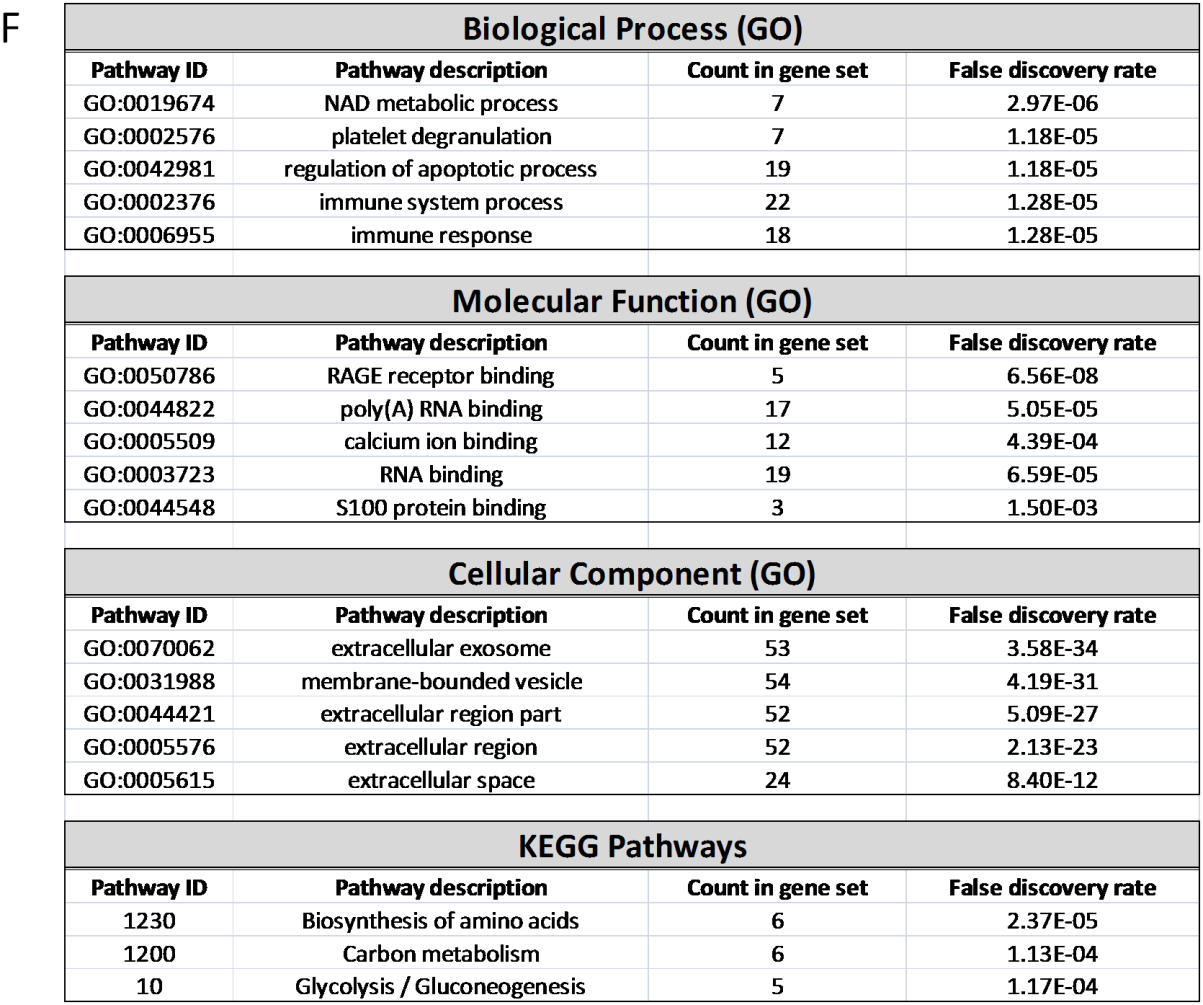
Proteomic correlations with S100 proteins **(A)** Prototype of a breast cancer tissue. Red asterisks indicate the 453 protein spots identified by MALDI-TOF mass spectrometry. The S100-protein spots are highlighted and the different isoforms labeled with alphabetical letters starting from the more acidic one. **(B)** Pie-chart showing for each S100 protein the percentage of the correlated proteins, among the 453 identified. **(C)** Pie-chart showing the number of protein spots correlated with single or multiple S100 proteins. **(D)** Histogram of 64 unique proteins showing significant correlation with at least five S100-protein spots. Black boxes represent the correlated proteins. **(E)** Interactome derived from the proteins listed in histogram 7D by using String database. **(F)** Tables reporting the 5 top significantly biological processes, molecular functions, cellular components and key pathways evaluated by using the String Analysis tool.

Taking advantage of our previous proteomic data [8, 20, 21], we performed a correlation analysis of the relative expression levels of each S100 protein spot identified in the proteomic maps (15 protein spots), with the global protein complement (453 protein spots identified by mass spectrometry) by using a pearson correlation statistical test. Figure 7A shows a proteomic map representative of breast cancer surgical tissue, where the 453 protein spots identified are marked with red asterisks and S100-protein spots are highlighted. Different S100-isoforms (S100A6 two isoforms, S100A7 two isoforms, S100A11 three isoforms, S100A13 two isoforms) are labeled by alphabetical letters starting from the more acidic one. As reported in Figure 7B, each S100 protein and isoform was correlated with a different set of proteins, suggesting distinctive roles for S100 proteins and isoforms. Specifically, among the 453 protein spots identified in our proteomic maps (corresponding to 271 genes), 236 protein spots (corresponding to 52% of the total proteins), were significantly correlated with at least a S100 protein spot. Figure 7C shows, in particular, the number of protein spots correlated with single or multiple S100 proteins. To give greater strength to the results we have chosen to carried out the functional classification of S100-correlated proteins with the protein spots, showing significant correlation with at least five S100-protein spots, listed in Figure 7D. Functional enrichments in the S100-correlated proteins were analyzed by using the String Analysis tool and clusterized through GO classification and KEGG pathways. Interestingly, the interactome analysis (Figure 7E) showed that the S100-correlated proteins contained more interactions among themselves than what would be expected for a random set of proteins of similar size, (number of edges: 285, expected number of edges: 111; PPI enrichment p-value:< 1.0e^-16^) indicating they were at least partially biologically connected. Again, the biological connection concerned the implication of the immune response, probably through the RAGE receptors signaling. Moreover, S100- correlated proteins affect NAD metabolic and apoptotic processes (Figure 7F).

### S100 expression in laser captured microdissection (LCM) microarray data set

**Figure 8:**
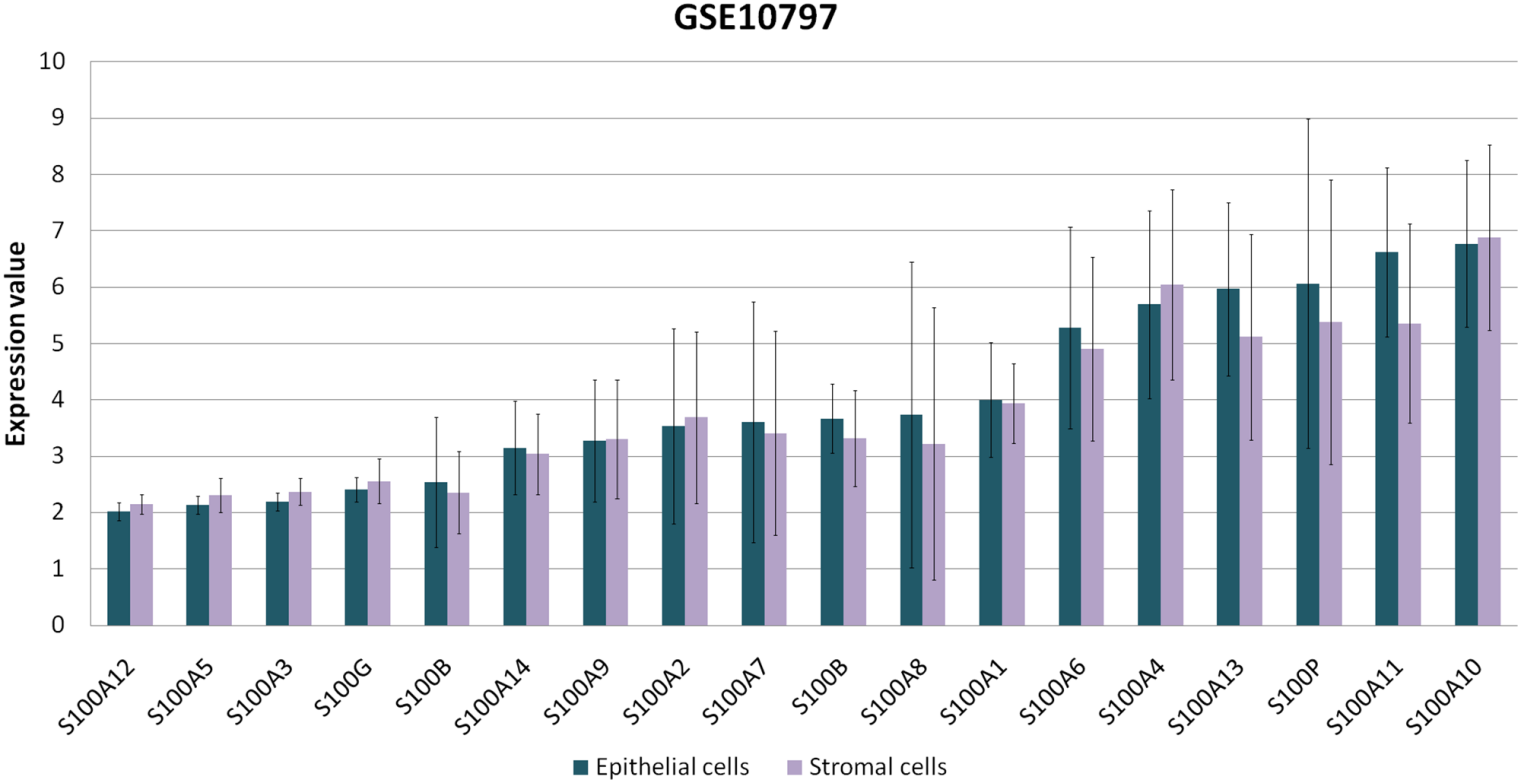
S100s expression values between epithelial and stromal cells Expression levels of S100 proteins derived from the available microarray data set (GSE10797) in GEO Dataset and analyzed by GEO2R tool.

In order to verify if S100s are expressed by epithelial cells, stromal cells or both, we analyzed the public microarray data set from GEO (GSE10797), containing the expression data from epithelial and stromal cells that were laser captured from invasive breast cancer (n=28) [25]. For each S100 probe, the expression values were analyzed by GEO2R tool. As reported in the Figure 8, no significant differences were observed between the epithelial and stromal compartments. As evident from high values of standard deviation, S100 expression levels are more variable between different tumors.

## DISCUSSION

Accumulating evidences have demonstrated that S100 family members play a critical role in cancer development. Several reports deal with the correlation or the involvement of individual S100 members in cancer [26–35], but their possible coordination and collective role, as well as the functional implications of their expression are still poorly known. In this study, we used a systematic multiomics approach to assess the role of S100 family members in BC. In comparison to normal tissues, the transcriptional levels of S100 members are robustly upregulated although in some cases a down regulation was found, indicating that S100 could act both as oncogenes or tumor suppressor genes, and exert both pro- and anti- tumorigenic actions depending on the tumor type. For example, the over-expression of S100A2, S100A3, S100A6, S100A8/A9, S100A11 and S100A14 have been documented in several cancer types. Conversely, under-expression of these proteins has been found in other cancer types [36]. Moreover, has been demonstrated that S100A2 expression is suppressed early during lung carcinogenesis [37].

By using cBioPortal database we verified if the deregulated expression of S100 family members in breast cancer could be caused by genetic alterations. Interestingly, the results showed that the predominant pattern of amplification (about 18%) occurred in the S100 genes clustering into 1p chromosome, while a low percentage of alterations that include both gene amplifications and gene deletions were recorded for the other S100 genes. Recently, it was reported that chromosome 1q21.3 amplification is a trackable biomarker and actionable target for breast cancer recurrence [38], so it is possible to explain these evidences also in consideration of the deregulated S100 expression.

We also investigated the prognostic value of S100 family members in BC by using KM plotter. Among them, 14 members were significantly associated with prognosis, evaluated as different survival, and only S100A2, S100A3, S100A13, S100A14, S100A7A and S100B were not associated with prognosis. These results are in agreement with the results obtained by Zhang et al., [35] about the prognostic values of S100 with the Overall Survival, and add new information about the individual S100 protein prognostic significance evaluated as Relapse Free Survival and Distant Metastasis Free Survival. Moreover, when the survival analysis was performed including all the S100 members, higher expression levels of S100s were significantly correlated with a shorter survival, indicating that S100 family members have collectively a greater prognostic value than the individual genes. Although the functions of S100 proteins have been extensively studied and the functional modes of S100 proteins can be intracellular, extracellular, or a combination of both [39], the molecular mechanisms by which S100 proteins contribute to cancer progression are not fully understood. The pathway and network-based analysis using data mining and a new correlation analysis with our previous proteomic data revealed that collectively S100-associated proteins are involved in relevant biological pathways correlated to immune response and inflammation. Our results are in agreement with many studies showing that, during infection, certain S100 proteins act as damage-associated molecular patterns (DAMPs) and interact with pattern recognition receptors to modulate inflammatory responses, [40, 41]. In addition, these inflammatory S100 proteins have potent antimicrobial properties and are essential components of the immune response against invading pathogens. Moreover, the creation of a pre-metastatic environment depends on the activation of inflammatory pathways [42] in the surrounding tissue, increasing the “mobility” of cells and thus facilitating the development of distant metastases or modulation of cell growth and differentiation [43]. So far, for several S100 members (i.e S100A7, S100A8, S100A9, and S100A12) a role in innate immunity has been demonstrated [44–46], while for S100A15, which is highly homologous to S100A7 (93% identity), a role in innate immunity is proposed as well [47]. Moreover, the association of S100 with autoimmune diseases has been known since long time in different inflammatory conditions. For example, S100A4 is involved in autoimmune pancreatitis [48], S100A11, S100A8 and S100A9 in rheumatoid arthritis [49, 50], S100A1 in hypoxia-induced inflammatory response in cardiomyocytes [51], S100A12 in Crohn’s disease [52], S100A7 and S100A7A in psoriasis [53], S100A8, S100A9 and S100A12 in systemic lupus erythematosus [54]. Nevertheless, the exact mechanism leading to S100-induced inflammatory reactions has not been identified. Probably, the binding to specific receptors, RAGEs (receptor for advanced glycation end products) and Toll-like receptors (TLR4 and TLR2) mediates the proinflammatory axis. Infact, upon stimulation with proinflammatory cytokines S100 proteins have been shown to be strongly upregulated, and in turn, S100 upregulation determines the overproduction of cytokines and interleukins [54–56]. This proinflammatory microenvironment promotes the secretion of various cytokines and growth factors into the tumor microenvironment. Recently growing evidence suggests that the tumor microenvironment plays a central role in the promotion of tumor metastasis [57]. S100 proteins, infact, can be considered as inducer of a cytokine network enabling tumor cells to engage angiogenic and migratory pathways [55], modulating the ECM molecules and the ECM dinamycs. For example, extracellular S100A4 stimulates invasive growth of mouse endothelial cells and modulates MMP-2, MMP-9 and MMP-13 matrix metalloproteinase activity [58, 59], while S100A7 modulates MMP-9 expression [60]; S100A14 protein is involved in cell invasion by affecting the expression and the function of matrix metalloproteinase MMP-2 [61]. Interestingly, by using the expression data from LCM between cancer epithelial and stromal cells we verified that S100 proteins are expressed in both cancer and stromal cells. Elucidation of these mechanisms would have a significant impact on understanding the pathogenesis of inflammation-associated tumors and in particular of breast cancer, and could aid progress in the development of more effective cancer therapies.

## MATERIALS AND METHODS

### ONCOMINE database analysis

ONCOMINE (http://www.oncomine.org), is an online microarray database, able to analyze the mRNA expression differences between tumor and normal tissues in common human cancers. For each cancer and gene, the thresholds were set as follows: p-value: 0.01; fold change: 2; gene rank: 10%; analysis type: cancer vs. normal analysis; data type: mRNA.

### GOBO database analysis

GOBO database (http://co.bmc.lu.se/gobo), allow a rapid assessment of gene expression levels, identification of co-expressed genes and association with outcome for single genes, gene sets or gene signatures in an 1881-sample breast cancer data set, generated on Affymetrix U133A microarrays [55].

### Kaplan-Meier Plotter database analysis

The KM Plotter database (http://kmplot.com/analysis/), able to assess the effect of 54,675 genes on survival using 10,461 cancer samples, including 5,143 breast, was applied to evaluate the prognostic values of S100 family members in breast cancer [24]. The desired probes ID were entered into the database by using the multigene classifier. Patients were splitted into high and low expression group by the median values of mRNA expression or by the best cut-off, as indicated.

### cBioPortal database analysis

The cBioPortal (http://www.cbioportal.org) for Cancer Genomics provides visualization, analysis and download of large-scale cancer genomics data sets [56].

### STRING database analysis

STRING (https://string-db.org) is a database of known and predicted protein-protein interactions. The interactions include direct (physical) and indirect (functional) associations; they stem from computational prediction, from knowledge transfer between organisms, and from interactions aggregated from other (primary) databases analysis [57].

### Proteomics of breast cancer tissues and correlation analysis

Proteomic analysis was performed on 100 breast cancer tissues following surgical interventions during the years 2003–2007 at the “La Maddalena” Hospital of Palermo, as previously described [8]. Briefly, surgical samples were homogenated overnight at 4 C with RIPA buffer, containing 50 mM Tris pH 7.5, 0.1% Nonidet P-40, 0.1% deoxycholate, 150 mM NaCl, 4 mM EDTA and a mixture of protease and phosphatase inhibitors (0.01% aprotinin, 10mM sodium pyrophosphate, 2mM sodium orthovanadate, 1mM PMSF). After centrifugation, the obtained supernatant was dialyzed against ultrapure distilled water, lyophilized and resuspended in ISOT buffer (4% CHAPS, 40 mM Trizma base, 65 mM DTE and a trace of bromophenol blue in 8 M urea). Aliquots containing 45 μg of total proteins were rehydrated in rehydratation buffer containing 8 M urea, 2% CHAPS, 10 mM DTE and 0.5% carrier ampholytes (Resolyte 3.5–10). The first electroforetic separation of 2D-IPG was performed on 18 cm long strips with a pH range 3.0–10. The strips were then equilibrated in a solution containing 50 mM Tris-HCl pH 6.8, 6 M urea, 0.5% SDS, 30% Glycerol, 130 mM DTE and 135 mM Iodoacetamide and then separated on 9–16% linear gradient polyacrylamide gels (SDS-PAGE) with a constant current of 20 mA/gel [58, 59]. The gels were silver stained and analyzed with the dedicated ImageMaster 2D Platinum software. Protein identity was assigned by peptide mass fingerprinting using the Voyager DE- MALDI-TOF mass spectrometer as described [7, 9, 60, 61]. The expression level of the protein spots were calculated as the volume of the spots (i.e., integration of optical density over the spot area), relative to the sum of the volume of all spots on each gel (%Vol). Measurements of relative expression levels of individual protein spots were normalized in each proteomic map for actin content (N%V), as previously reported [20]. Correlation analysis with the collective profile of cancer patients proteomics was performed using the Pearson correlation test. Correlation coefficient ≥0.4 and p <0.05 was considered significant.

## CONCLUSIONS

This study confirmed the prognostic value of mRNA expression of the S100 family members in breast cancer and pointed to the molecular mechanism through which S100 affects cancer progression, probably regulating innate immune response and inflammation pathways. The extracellular activities of S100 proteins depend on the cell-specific expression patterns, the specific targets and the local microenvironment as well. For the first time, an integrated multiomics approach performed on S100 family members, allowed to extrapolate new insight regarding the collective role of S100 in BC. Interestingly, although is known that the correlation between mRNA and protein abundances are often poor in the cells, by using large-scale dataset derived from transcriptional and proteomic data, we obtained a good convergence between S100-regulated pathways. Further studies will be necessary to understand the role of epigenetic changes and the different protein-isoforms.

## SUPPLEMENTARY MATERIALS TABLES








