# Supplementary material for: A multiomics analysis of S100 protein family in breast cancer
Source: Oncotarget. 2018 Jun 26;9(49):29064–81. doi: 10.18632/oncotarget.25561 (PMC6044374; doi:10.18632/oncotarget.25561)
Supplement: Supplementary file 1 [file oncotarget-09-29064-s001.pdf]

A multiomics analysis of S100 protein family in breast cancer

SUPPLEMENTARY MATERIALS

Supplementary Table 1: List of interacting proteins from STRING

See Supplementary File 1

Supplementary Table 2: List of interacting proteins from GOBO

See Supplementary File 2

Supplementary Table 3: List of interacting proteins from ONCOMINE

See Supplementary File 3
