# Supplementary material for: A multiomics analysis of S100 protein family in breast cancer
Source: Oncotarget. 2018 Jun 26;9(49):29064–81. doi: 10.18632/oncotarget.25561 (PMC6044374; doi:10.18632/oncotarget.25561)
Supplement: Supplementary file 2 [file oncotarget-09-29064-s002.docx]

| **Supplementary Table 1:**  **LIST OF INTERACTING PROTEINS FROM STRING** | |
| --- | --- |
|  |  |
| **S100A1** | TLR4; HSP90AA1; LY96; HSP90AB1; PPID; FKBP4; HSPA4; ANXA6; S100A2; HSPA1B; HSPA1A; PLN; HSPA1L; HSPA2; S100A13; GFAP; SLC39A4; HSPA8; HRAS; HSPA6; TP53; TOMM70A; SPRR3; DES; S100A7; RYR1; HSP90B1; CACYBP; S100A4; ATP2A2; GLI1; SPRR1B; AGER; MYZAP; TCHH; CALM2; CALM1; CALM3; CAPZA1; |
| **S100A2** | TP53; TP63; S100A1; S100A7; EGF; KRT6A; GDF15; MDM2; PPID; FKBP4; KRT5; S100A8; C17orf105; |
| **S100A3** | S100A7; PLCB3; |
| **S100A4** | MYH9; CDH1; TP53; TOM1L2; ZNF526; SNRPF; ANXA2; MYH2; NFAT5; S100A6; VEGFA; LAMTOR5; SPP1; MMP13; FGF2; ERBB2; AKT1; BNIP3; VIM; SMAD2; EGF; SMAD3; SRC; SELENBP1; SDF4; FGF13; PSMA1; IGBP1; UNK; MMP11; PGLYRP1; IFNG; EFNA1; CDKN1B; AKTIP; MDM2; RHOA; ACTA1; NOV; MYH10; MYH4; DDX5; SNAI1; MMP9; PPFIBP1; S100A7; S100A1; CD164L2; TPM3; TGFB1; HBEGF; EGFR; RBPJ; MYH14; STRC; CIITA; TIMP1; S100A8; MYCN; AREGB; SNAI2; MMP2; METAP2; AREG; |
| **S100A5** |  |
| **S100A6** | AGER; ANXA2; CACYBP; ANXA11; S100A10; SEC24A; TMSB10; TP53; TCHH; S100A4; S100A8; GAPDH; FLG; IVL; CALM2; CALM1; CALM3; FKBP4; PPID; LOR; SPRR1A; CD2BP2; NUP214; CLNS1A; DHX36; TOM1L2; SEC23A; C21orf59; LGALS3; RB1; SPRR3; CTNNB1; ANXA1; PGR; ATXN3; SDF4; RELA; CRH; HRAS; BAMBI; SPRR1B; FKBP5; TPR; HEBP1; C4BPA; ZNF395; S100A11; S100B; |
| **S100A7** | MYC; BRCA1; TBP; MAX; COPS5; FABP5; RANBP9; RNASE7; S100A9; S100A12; DEFB4A; S100A8; IL17A; DEFB103B; DEFB103A; EGF; S100A2; CAMP; DEFB1; MMP9; VEGFA; IL22RA1; ADSL; IL22; XCL1; S100A1; S100A11; S100A4; KRT16; S100A3; IL10RB; AMY2B; RASSF7; IVL; TCHHL1; |
| **S100A8** | S100A9; TLR4; LY96; MYD88; TIRAP; S100A12; NCF2; IL1B; PTPN11; CDH1; MNDA; IL8; SRC; BECN1; TGFBR1; GAPDH; FPR1; S100A7; FCGR3A; S100A6; MARK2; MET; HPGDS; TLR2; GRB2; CSF3; HGF; SMAD4; ATP4A; ATP12A; IL6; BAX; F11R; GAST; TNF; SMAD3; SMAD2; BIRC3; S100A11; IL10; PRDM10; DMWD; DNMT1; LILRB2; ALB; HCK; FCN1; MIF; MGMT; AQP9; CAMP; CTNND1; LCN2; VNN2; ITGAM; MMP9; KIAA1524; TP53BP2; SMAD7; CXCR2; FOXM1; TP53; TGFB1; DDX53; INHBE; S100A2; EFNA1; MBOAT4; JUN; NOS2; CD79A; SERPINE1; ITGA5; TAOK1; GADD45B; CCL2; ANKRD1; LCE3B; S1PR4; LCE1A; PKN2; RAB10; GAB1; ARHGEF6; PTGS2; S100A10; S100A4; CD14; MPZL1; IL1RN; HRAS; LYZ; TYROBP; EGF; IL17A; IL4; NOD1; NFKB1; ITGB2; ANKRD2; |
| **S100A9** | S100A8; LY96; TLR4; MYD88; TIRAP; S100A12; TOP1; SAP18; S100A7; IL6; IL1B; NCF2; TNF; MNDA; FPR1; PPP2R1A; PPP2R2B; TLR2; SF3B14; DEK; SRSF11; FCGR3A; STAT3; GAPDH; CD68; IVL; GLI1; LCN2; NCF1; VTN; VNN2; LUC7L3; TLE3; PSRC1; MATR3; SRRT; LILRB2; IL8; LYZ; CD14; TYROBP; HNRNPU; HRAS; AQP9; HCK; MYB; DSTN; SRSF5; NCSTN; SSSCA1; U2AF2; DDX21; TOP2B; NUBP2; U2AF1; SRSF3; SART1; CD2BP2; PSMA5; MIF; DHX15; FCN1; MMP9; CAMP; ACIN1; SON; SRSF7; RBM14; PRPF8; PSMB7; UTP14A; TPR; CD163; IL10; SDF4; ITGAM; UNK; SUPT16H; SRSF10; CPLX1; UBC; THRAP3; TRIM55; SMARCA5; TRA2A; RBM39; TRA2B; ZC3H18; CXCR2; TPBG; GTF3C1; FLT1; GLIS1; ZNF326; LCE3B; LCE1A; GADD45B; SURF4; RNPS1; SSRP1; NOS2; CCL2; DCLK1; MPO; SPRR1B; |
| **S100A10** | ANXA2; PLG; PLAT; KCNK3; S100A6; ANXA2R; TCHH; SCN10A; TRPV6; FLG; IVL; LOR; PLA2G4A; ANXA1; DLC1; TMEM65; SPRR1A; BAD; SPRR3; TRPV5; SPRR1B; C4BPA; AHNAK; ATP6V1E1; HDLBP; SRPRB; 0.505; SIN3A; ELAVL1; FLOT1; ZMPSTE24; UBAP2; CD55; SRP9; WDR92; ZFR; FAF2; PHF5A; ANXA7; CFTR; RPL10A; CTSB; RPL10L; PPIF; PRNP; RPL12; S100A11; HTR1B; PLAUR; S100A8; GP9; GTPBP1; |
| **S100A11** | ANXA1; ANXA2; NCL; AGER; PLP2; STAM; IGBP1; TACC1; HDLBP; UBE2F; LASP1; S100A8; RAD54B; AKT1; SP3; ACTB; S100A7; S100A10; BTK; S100A6; |
| **S100A12** | AGER; HMGB1; NFKB1; MAPK3; APP; SAA1; MAPK1; RELA; S100B; NFKB2; CAPZA2; CAPZA1; S100A8; S100A9; VNN2; S100A7; GGT1; GGT2; GGCT; FCGR3A; FCN1; IL18RAP; CEBPB; FPR1; NOX1; PROK2; QPCT; GCA; PSMC6; FCGR1A; |
| **S100A13** | FGF1; IL1A; PBRM1; CRABP2; SYT1; S100A1; RARB; STK4; NUDT21; THOC7; NUCB1; |
| **S100A14** | S100A16; DLEC1; RNF6; LZTS1; NUCB1; ADH1B; RAB25; ALDH2; ATP8A2; CDH2; KLF4; WWOX; |
| **S100A7A** |  |
| **S100A16** | VAPB; C8orf40; SUCLG1; SUCLA2; TMEM9; TMED10; IDH3A; OXCT1; S100A14; TCOF1; STIM1; CPNE2; UBA52; SUCLG2; HNRNPA2B1; STMN1; TOR1AIP1; MPST; MSN; RTN4; SGSH; TMEM177; XRCC1; TST; SNRPA1; VAMP2; UBXN1; |
| **S100B** | GFAP; APP; AGER; HMGB1; CAPZA1; NFKB1; MAPK3; MAPK1; RELA; NCOR1; NFKB2; SAA1; S100A12; CAPZA2; TAB2; FGF2; ERVW-1; AKT1; CXCR4; TP53; ITIH4; BST2; RPS6KA1; CCR5; MAPT; ACOT8; RPS6KB1; GUCY2D; CALM1; HLA-A; HCK; CALM3; CALM2; SRC; LCK; ENO2; AHNAK; STK38; IL2; CCL5; ANXA6; SOX9; VAV2; IL1B; PACS2; GUCA1A; CD69; PACS1; RPS6KA3; NMT1; FASLG; BCL2; EGF; APOE; CCL2; ICAM1; RPS6KA2; PAK2; SOX10; APOBEC3G; PIK3CD; PIK3CG; PIK3CB; TRIM5; DICER1; PIK3CA; PI3; CD289; TLR9; ANXA5; CTLA4; DNAJB1; DNM2; RBM8A; ZAP70; LYN; CFL1; INSL3; ABCA1; MLL5; MAP3K5; VAV1; RPS6KA6; MAP2; S100P; MAPK14; SLC5A5; SCARB2; CAV1; ALB; PRDM10; MBOAT4; ENPEP; TBK1; FPR1; TNF; CTNNB1; IL6; SCARB1; CD36; STK38L; RAB11A; TNFSF13B; ATP6V1H; ARF1; SYNPO; HLA-B; PAK1; MRAP; TJP1; RPS6KB2; BECN1; JPH2; AP1M2; SOCS1; MBP; MMP13; PNMA2; NFATC1; MYH9; MDM2; SRSF1; CCNT1; NES; SDF4; BDNF; GAPDH; TXNIP; CACYBP; DES; CCNK; STEAP4; TPP2; CYBRD1; ARHGAP35; RBFOX3; FGF9; IL10; HLA-C; IQGAP1; CD209; VSNL1; DDX41; APOBEC3F; INS; S100A6; LY86; FGF13; |
| **S100G** | TRPV6; CALB1; TRPV5; VDR; SDF4; PVALB; PTH; ATP2B1; CABP2; CYP27B1; AFP; CA7; FGG; APOH; SLC8A1; SI; DNAH8; CALM1; CALM3; CALM2; CYP24A1; |
| **S100P** |  |
| **S100Z** | S100PBP; SNRPF; CA8; NUSAP1; IGBP1; S100B; PSMD4; LRRC45; ZNF526; PPM1G; PRAM1; CEACAM6; |
