# Supplementary material for: A multiomics analysis of S100 protein family in breast cancer
Source: Oncotarget. 2018 Jun 26;9(49):29064–81. doi: 10.18632/oncotarget.25561 (PMC6044374; doi:10.18632/oncotarget.25561)
Supplement: Supplementary file 3 [file oncotarget-09-29064-s003.docx]

| **Supplementary Table 2:**  **LIST OF INTERACTING PROTEINS FROM GOBO** | |
| --- | --- |
|  |  |
| **S100A1** | SCRG1; SOX10; MFGE8; |
| **S100A2** | KRT5; KRT17; KRT14; DSC3; DSG3; SERPINB5; TRIM29; MMP7; KRT6B; LAMB3; CDH3; GABRP; SCHIP1; KRT6A; ACTG2; MIA; PLS3; KLK6; ADORA2B; LAMC2; FAT1; KRT16; ACTN1; MFGE8; BBOX1; FZD7; VSNL1; |
| **S100A3** | DAO; PIM1; B4GALT2; |
| **S100A4** | TGFBI; TYROBP; NNMT; LGALS1; EMP3; VIM; RARRES2; GMFG; NCF2; LY96; CLEC2B; CD14; NT5E; TUBB6; CCR1; LYN; PLTP; GPNMB; CTSL1; MNDA; EVI2A; S100A10; HLA-DMB; ITGB2; C1R; TIMP2; PRKCDBP; VSIG4; ANXA1; FCGR2A; SERPINF1; SLC1A3; CD97; SLA; HCK; FCER1G; ARPC2; CTSK; CORO1A; CTSB; C3AR1; GSTO1; LAPTM5; WIPF1; C1S; NPC2; FABP5; IL32; VCAM1; IFI16; ADCY7; C1QB; FCGR2B; GLIPR1; MGAT1; SRGN; CPVL; CD53; PLS3; NCF4; ARPC1B; CD86; CYBB; DOCK2; DPT; HLA-DPB1; LCP2; TLR2; ERBB3; DLG3; |
| **S100A5** |  |
| **S100A6** | C11orf75; SPHK1; ACOT9; CISD1; REXO2; PQLC1; KCTD9; PDXK; CHIC2; DOK5; IGF2BP2; CCDC109B; C19orf60; MYL12B; TRMT116; TMEM43; CD58; FNDC4; YEATS2; FXYD6; KRT81; SOD2; TMBIM1; TMEM45A; DUSP22; C1GALT1; ANKRD27; WBP5; ARGLU1; MTCH1; DSE; TRIT1; EXOSC8; |
| **S100A7** | S100A9; S100A8; LCN2; ASS1; CLCA2; KYNU; |
| **S100A8** | S100A9; S100A7; RARRES1; KYNU; C1orf106; PI3; CEBPB; ASS1; LBP; NFIL3; FUT3; CCL18; LCN2; MMP1; PFKP; HIF1A; SLPI; FAM134B; DNAJC12; SALL2; IL6ST; MAPT; CLSTN2; GATA3; MYST4; CA12; STK32B; TBC1D9; IGF1; STC2; ESR1; SCUBE2; KDM4B; |
| **S100A9** | S100A8; S100A7; RARRES1; ASS1; KYNU; LCN2; NFIL3; CEBPB; HIF1A; KMO; SLPI; MMP1; ORM1; PI3; LBP; NP; SOX11; AKR1B10; PFKP; APBB2; MAPT; IL6ST; NEDD4L; ESR1; TBC1D9; KDM4B; STC2; |
| **S100A10** | LGALS1; TGFBI; NNMT; S100A4; SERPINH1; ARPC2; VIM; TUBB6; PLIN2; S100A11; ADM; ANXA1; NECAB3; SFRS12; MYST4; ESR1; LRBA; SLC22A5; NEK4; |
| **S100A11** | POR; TAGLN2; PFN1; SDC1; ARPC1B; SLC5A6; S100A10; VPS72; EFNA4; BCL2; |
| **S100A12** | CPA2; DAO; DRD5; CPA4; CCDC9; FASLG; CD80; HECW1; SMCP; C7orf28A; NR6A1; OR10H3; SLC6A12; TUB; TGFA; ERCC6; INHBC; KLRG1; CA1; XDH; KRT2; PMS2L11; DLX4; IPO13; FANCC; GML; GRM6; LECT1; FGF5; POU4F2; CA7; TNFRSF13B; ACRV1; CPN1; NEUROD2; ABL2; CYLC2; HABP2; KRT3; GP1BA; SEC14L5; ZNF536; |
| **S100A13** |  |
| **S100A14** |  |
| **S100A7A** |  |
| **S100A16** |  |
| **S100B** | SFRP1; SOX10; CRYAB; MIA; ID4; GPM6B; FAM107A; WIF1; SCRG1; GABRP; |
| **S100G** |  |
| **S100P** |  |
| **S100Z** |  |
