# Supplementary material for: A multiomics analysis of S100 protein family in breast cancer
Source: Oncotarget. 2018 Jun 26;9(49):29064–81. doi: 10.18632/oncotarget.25561 (PMC6044374; doi:10.18632/oncotarget.25561)
Supplement: Supplementary file 4 [file oncotarget-09-29064-s004.docx]

| **Supplementary Table 3:**  **LIST OF INTERACTING PROTEINS FROM ONCOMINE** | |
| --- | --- |
|  |  |
| **S100A1** | MRAS; CRYAB; ROPN1; FABP7; MICALL1; LARP6; |
| **S100A2** | ATF4; BNC1; COL4A6; ITGA6; SLC6A15; FLRT3; STIL; LAMC2; LAMB3; ALDH3A1; ACTN1; COL7A1; SMARCD1; GM2A; SULT1E1; TMPO; COL4A5; POU2F2; ADRB2; FEZ1; SLC20A2; PSMD3; HOXA1; EPHX1; DCBLD2; L1CAM; ITGA2; TGFA; NFE2L1; SLC9A1; SVEP1; CYP3A4; MCC; |
| **S100A3** | HRASLS2; CRHR1; TPI1; ELAC2; KCNH6; XKRY; AOX1; PLA2G1B; MC2R; NPHP4; MAPK9; ZNF695; P2RY10; FRK; BCAS1; ALK; PDE12; KLRB1; PTN; KRT36; KLRD1; HDC; PCDH7; TLR7; ITGA8; PTGS1; DNAH3; TEF; ADD2; CASP8; RPS6KB1; TRAPPC9; MT3; C11orf1; ITGA2B; HLA-DPA2; MTAP; TIPIN; CD40LG; KIR2DL4; C1orf114; WNT6; IGFALS; AGRN; ESR2; TCF3; SLC8A2; FGF18; PCDH11Y; C7orf28A; PPP3CC; MCHR1; HOXD3; ADRA1D; NROB1; CCR4; KRT13; MYH15; CDS1; FGF5; PDX1; RPL21P2; SLC22A8; |
| **S100A4** | AP1S2; ELK3; HSD17B11; AEVI2A; SH2B3; EMP3; AXL; THBD; NRP1; GPR116; GNG11; G0S2; CCRL2; NRGN; C9orf167; TMEM22; SLC43A3; AKAP2; PALM2-AKAP2; PRKCA; SLC9A6; NPC1; AKR1B1; DFNA5; CHST7; SYDE1; TRAM2; COL13A1; EHD1; STK17A; RHOF; CD97; RAC2; PLAC8; PLIN3; VEGFC; ARHGEF18; EFHD2; SLCO4A1; HPCAL1; FOSL1; TBC1D13; ARL2; CCDC85B; BMP4; HBEGF; NRG1; ACSL5; S100A5; WNT5B; ZNF215; CSF2; CDA; FOXF2; FLNC; ANKRD1; IGFBP1; OSBPL10; CDCP1; HMGA2; F2RL1; SHC3; MORC4; DNMBP; IL1RAPL1; C4BPB; PPP1R10; FADS1; CLTB; ZNF185; PRSS3; ADRB2; GLTP; |
| **S100A5** | ARHGAP23; SPI1; CHD8; CNTF; MYH13; KCNJ1; HOXA1; GJA5; GNG2; ORF1; LOC148696; NFASC; OLFM2; CADPS; GAS7; SLC26A6; PDE6G; GRIK2; TBCID19; NMT1; CAND1; OGG1; ATP2A1; DLX3; POLN; SLC22A11; GPLD1; CHRNB1; MAP1A; CRHR2; CACNA1H; NEURODH; HTN3; LCAT; QPCLT; TFR2; PIGN; IDUA; CAV3; MLNR; CACNB3; KCNG1; AQP8; RAD51L3; ACE; UNC93A; USH2A; ARRB2; SNX12; MAD1L1; PODXL2; GNA13; OR7A5; TIMM22; MAN2A2; TAOK2; ITSN1; TMEM59L; P2RX6; NIPAL3; FANCF; NKX3-1; POLM; INTS9; NBEAL2; OBP2A; LOC731157; AGTRAP; PPME1; RAB35; HOXC4; ANKRD16; HTR7; EFCAB6; CBARA1; EHMT2; RLN1; IPO8; PGBD3; THRB; TBC1D22A; GRK1; MTAP; ZNF461; ADORA1; FAAH; CDK5; STMN3; SRY; CSHL1; MGAT3; MAG; HIST1H2BL; TNPO2; C21orf2; NRP2; GALK2; MYO5A; MKLN1; PSTPIP1; SLC22A17; GPX2; DKFZP434K028; PGLYRP1; IFRD2; THOC5; GOSR2; ATG7; CHP; FFAR2; TGM2; CWC27; ITGB3; CCDC76; MTRF1; FAM48A; SULT4A1; RDH5; SFTPD; ACHE; GATA4; SGTB; NTF3; OPRD1; OBP2B; MS4A12; GMIP; A2BP1; RRH; GRAP; PDE4DIP; HYAL1; BTRC; AK3; TMOD3; IDE; LNPEP; ZAK; DNAJC4; FAIM2; CDC42EP2; CCDC22; STX8; CEPT1; RERE; NEU3; TNNT3; ARNT; C1orf114; AGPAT4; SMPD3; FAM70A; TBX6; TRMT1; KCTD5; IRAK4; C12orf29; ANAPC10; GCNT3; ARID3A; DLG3; CS; ARFGEF2; SMPD2; HPCAL1; PIK3CB; OSBPL3; PRKDC; RASAL2; FLAD1; PRPF3; CHRNB4; ELAVL3; ICAM4; KLKB1; KCNJ2; HRH1; HTR1F; INPP4A; SMARCD3; TULP4; KCNK6; SLMAP; NIN; C10orf137; COX15; UBE2G1; TRIM33; KIAA0895; SLC6A2; CRK; ZNF236; MTMR9; CHMP2B; EIF5B; ATRX; CDK20; SLC22A23; PPA2; SUOX; NFS1; ELAVL2; C14orf79; ARRB1; SPTBN5; ADRB1; SUPT3H; MAP3K6; HES1; HIPK2; DLG5; TRAF3; SH3BP2; CBLC; GAL3ST1; TFAP2A; GABRA3; USF1; DIRAS2; FOXI1; ARKGEF10; THBS3; CC2D2A; |
| **S100A6** | SYNM; BBS7; TMEM65; KCMF1; ENAH; FBXW7; GPR161; LRIG3; NCRNA00085; FAM57A; BEND6; RPS6KA3; PAPSS2; SEL1L3; ARPC1B; DAPK1; KLHL2; RNF145; HIVEP2; CORO1C; CSRP2; NHSL1; KIAA1804; MID1; UPP1; MPZL2; TLCD2; CA13; LOC401074; SEMA3E; CAMTA1; EGFR; INPP1; RCAN1; PHLDA1; GUCY1A3; VSIG10L; FERMT1; UBASH3B; C5orf46; TUBA4A; LHX2; L1CAM; SLC4A11; LOC653602; FAM84A; KIF1B; PIK3R1; FAM89A; ADM; ATP2C1; TMX4; TRIM2; PCDHA; PCDHA12; LY6K; |
| **S100A7** | S100A9; S100A8; |
| **S100A8** | DTX4; STC1; TMPRSS2; CES2; PIK3R1; GABRE; PCDHA12; DKK1; DEFB1; CRISP3; PON3; S100A9; S100A7; IRX4; MDK; P2RY6; SCEL; ALOX15B; CLCA2; CP; FGG; KYNU; LBP; FAM49A; TMX4; NEB; ACE2; CAMK2G; PSACA; EPHB6; ADA; DNYC1I1; DDX43; CPA4; CTAG2; DPYSL4; FERMT1; CLCN4; CTAGG1A; PLEKHM2; HOXA9; ST14; CLU; HDAC9; MYOB1; SAA2; CITED2; GHADH; HNMT; GLB1L2; CASP4; RFTN1; ABCF2; PDE4A; MAN1B1; HYI; PCSK1N; BIN1; GRP87; AKR1B10; PRPF40A; ZBED1; GYG2; NTRK2; SQSTM1; SGGB2A1; GALNT14; GOS2; RBP4; FABP4; TLR2; ORM1; TTC38; C19ORF60; STAP2; HOOK2; FBXO42; LRP3; LHX2; RAB40B; HHIPL2; ENDOD1; TH; ORM2; ATP2C1; DEF8; COL6A1; SHOX2; CARKD; FAM89B; ARHGEF4; SLC10A3; C14ORF147; RFC5; IRAK1; MCAM; DAB2; LYN; HMBS; ANXA1; TGFB1; TFRC; EPAS1; STEAP1; BIRC3; PTGS1; SNX19; TMEM184C; TPGS1; TNFSF13; NSFL1C; MRPL4; SRPR; GPNMB; SLC31A2; TRIM68; EIF1AX; CDC37; PCDH7; TACC1; CD248; FAS; APOBEC3F; LGALS9; |
| **S100A9** | S100A8; S100A7A; S100A7; |
| **S100A10** | CAPN1; RBM6; UBTF; ARF5; ABLIM1; ACP1; RARG; PAFAH1B3; BLMH; THY1; DYRK1A; AMP2; IGF2R; GZMM; POLA2; TSC22D3; UPK2; DMWD; MOGS; ZAP70; STIP1; FSCN1; EIF4A1; MYL6B; METAP1; TTLL12; PPP2R4; UPF1; PPP1R11; DDR1; ERBB2; |
| **S100A11** |  |
| **S100A12** | SLC4A1; EPB42; HBM; AHSP; GATP; PADI4; TAL1; KLF1; NFE2; GYPB; RHD; GYPA; HEMGN; RHAG; TRIM10; TUBB1; RGL4; RNASE2; EPX; PGLYRP1; MPO; PRTN3; AZU1; DEFA4; ELANE; RETN; PRG3; MS4A3; CA1; MMP8; BPI; PAD14; CEACAM8; RNASE3; PRG2; CLC; MGAM; CLEC12A; CDA; CTSG; NCF1; CFP; MYL4; FCN1; IGLL1; EPB49; SLC25A37; PPBP; PF4; RFESD; CD300A; YPEL4; TMCC2; OSBP2; TSPO2; IL18RAP; ITG4A; RHCE; SERPINB10; GFI1B; CD177; TIMD4; LOC643332; SPTA1; GF1B; C19ORF59; KCNH2; PROK2; LILRA2; SNCA; CXCR2; GYPC; HBQ1; ITGB3; DEFA1; HMBS; CR1L; EPOR; FAM46C; VNN2; PLAC8; OLFM4; CRISP3; MAP2K3; XPO7; CLEC3A; BST1; IL6R; BNIP3L; GMPR; JAK2; DARC; RCSD1; SELL; RFN123; UBAC1; PDK4; TNS1; PLCL2; RAC2; ZEB1; EWSR1; TIMP4; ANK1; HBG2; FYB; PTPRC; SLC2A14; CSF3R; TPP1; PIP4K2A; ARRB2; FCGR3B; ARHGDIB; LILRB2; GRP84; FGFR1OP2; CERKL; CD300LF; SLA; FPR1; PRKCB; ALOX5AP; ACAP1; ITGAL; CYBB; SASH3; DOK3; MYO1F; FGR; SPI1; IL2RG; LST1; CD37; HCK; ITGB2; C16ORF54; NKG7; CST7; NCF1C; LYL1; NCF4; ARHAGAP9; BIN2; HK3PRAM1; CORO1A; RGS18; ATP8B4; MPP1; MNDA; LOC150166; |
| **S100A13** | RAD9A; SLC25A2; S1PR2; TMEM171; MGST2CDH5; SPRR2C; TRHH; FCRLA; KCNMB3; IL1F8; KLK2; THEG; GLYAT; CCDC113; LPHN1; RIT2; HDAC4; PNPLA6; OR2T2; ZC3H4; TRIM41; NLRP10; RAD51L1; LOC283701; FAM49A; ZNF253; OXCT2; BEST3; SP100; APOL4; HIST4H4; CPN2; C4ORF23; CHP2; NPB; ASB16; RFC2; PRICLE3; CYP2B6; PPID; PSPH; ADIPOQ; ANKDD1A; C3ORF51; SYNPO2L; IL4I1; ZNF62; ORC6L; FLJ11710; POLR2J2; PLCD3; SLC27A1; ZNF713; TMEM150A; CBX2; ITGAM; PALM2-AKAP2; STMN4; SLC6A19; VHL; ARHAGEF15; PLDAP; RUNX2; MRC2; CCL23; RNF157; HOXA2; CCNT1; MMEL1; CPA5; AMBRA1; KLF4; BEST1; SERPINA10; CREB3L3; CPLX2; TMSB4X; MAGEC1; F7; CUX2; CLVS1; TKTL2; PCYT2; DTX3; AVPR2; TNFAIP8L1; ABP1; OR1J1; GPR64; C16orf52; MGC27345; CHRNA10; KCNN1; P2RX1; TP53AIP1; PAX4; REC8; TNFAIP8L3; PRODH; PROV; YIF1B; CD3E; PRB4; MAMSTR; SHISA5; TECRL; PCDHB9; WFIKKN2; ETV4; C16orf3; CD3EAP; HNF4A; CEBPB; MLLT1; GPHA2; OCM2; FAM120C; MMP28; TACR2; USP17L2; TSPAN12; FLT3LG; GNAZ; SELPLG; LOC100129434; CACNA1B; RIMBP2; LOC100288749; KLF16; SH2B2; MUC3A; PTCH1; EMILIN3; POM121; LRRC41; CA11; LOC729792; TSNARE1; NECAP1; MASP2; LELP1; SRCAP; KCNN2; KCNE1; RBPJL; LY9; OR8J3; PANX2; RASD2; C22orf31; FOXA3; RHPN1; MAPK15; DLX3; UCN3; SLC25A45; SCRT1; FAM123C; JMJD5; MADCAM1; C1orf69; UPK3A; C17orf99; SRGAP1; USP6; SH3RF2; OR5F1; GPR139; TANC2; TMEM89; ZC3H10; FIBCD1; PPP1R3; BPTF; PEA15; ODF3L2; SMCR5; CENPM; HIPK2; UPK2; GPR172B; LGALS12; SLC16A7; PRDM7; CT45A5; MMP15; KIFC2; PPP2R1A; SDS; POLR2A; SPACA3; ZMIZ2; LOC145694; MYOD1; MON1B; MUC2; LOC399851; TCTE3; DCAKD; ADAMTS13; CYB561D1; ZC4H2; CASP8; ZNF843; MEF2B; GAMT; DKFZP434K028; PIP5K1A; MFRP; OSCAROPN1LW; CEACAM7; SRD5A3; TMIGD1; TSPAN32; DTNB; FAM182B; CEACAM1; HOXB13; PELI2; EIF4H; LOC154822; C14orf79; PTGR2; WDR48; PIAS2; SH2B1; ADPGK; GNG4; SGK196; SLC6A6; R3HDML; GSG1L; TRIM74; OR6Y1; IQCF2; TRPM6; PAS2R13; LGI4; GYG2; SLC35EA; DSC2; SFTPA1; HPCAL1; UBE2QL1; LDB1; ZNF440; MAB21L1; ST18; LOC100133920; SMYD1; CROCCL2; RGMA; CD19; MRPL52; LCE2C; KIAA1751; ATP8B1; PFKFB3; TMCC2; WIF1; TAS2R19; COL25A1; PITX3; A2BP1; OR52P1P; HYAL1; ORAI3; LPP; AMZ2P1; KRT2; TSHB; NXT2; KPTN; MYCN; CACHD1; WDR91; CLCA4; TRPV1; GNG13; DDA1; C1orf183; SPRY4; ZIM2; SSX5; SLC14A1; FOXR1; TFAP2A; |
| **S100A14** | C11orf67; HLA-DQB1; POLB; TAP1; MRPL13; TRGC2; LRRN2; CACNG4; CHAF1B; CLNS1A; HSD17B1; IFIH1; TMC6; LCK; KMO; AMD1; IL2RB; LOC100293559; LAIR1; TARP; LILRA4; CCL5; PYCRL; MMP11; APOC2; GPR172A; CEACAM5; PIM2; APOL6; CYBB; CCT5; HOXC10; HCP5; GCH1; FMNL1; ISG20; LAPTM4B; UBE2C; GAB2; NARS2; C2orf54; PRODH; CD8B; RALA; C2orf72; ARAP2; PSMA6; ZBED5; IRF1; PPT1; TFRC; ALC8; SQLE; PCSK2; TYMP; GPSM3; MX2; GRB2; SIRPG; HN1; GBP1; POLR2K; LAP3; CD2; IGKC; SP110; WARS; CXCL9; CXCL10; STAT1; CXCL11; OAS1; CEACAM21; HLA-DRB6; SCGB2A1; SECTM1; KLRK1; C8orf4; GSPT1; SOX11; SC4MOL; NKX2-2; PRKCA; PRRX2; DIO2; S100P; DERL1; OAS3; IFI44; OAS2; MX1; ISG15; PLAC1; CANT1; NDUFC2; KPNA2; COPE; ATP2A3; SCD; OASL; GNLY; MGAT2; IKZF1; IGKV3-20; TRD; APOC1; IGL; UBE2D1; IGLJ3; IDO1; IGHV1-69; DRD3; CDC45; GGCT; CD24; EWSR1; PAX8; TACR2; RAD54B; VNN2; CAMP; AMACR; HSD17B6; CYTH1; CD7; IPGEF1; CUL5; BTN3A2; IRF4; TRCV9; ASPH; ATP6V1C1; ACLY; LCP1; TUSP10; ZNF706; PLA2G16; ORMDL2; RAP2C; FGL2; YWHAZ; ALOX5AP; MAN1A1; TRAF1; HMHA1; JAK2; SULF1; ABCA1; FN1; CD86; KNO; NCF1; SLAMF8; CD27; GZMA; MS4A1; FAM82B; ARHGAP25; SP140; PYCR1; SEL1L3; IL2RG; PTPN7; NUDT1; GFI1; HYAL1; PVALB; KIF4A; APOBEC3B; KCNH6; RGS4; ERAP1; RET; BUB1; PRAME; CALCA; KIR3DL2; PDK1; LRIT1; BTG4; CNTNAP2; CXCR6; PITX1; CCNE2; CYP27B1; CENPE; TUBB; NCAPH; DEGS1; LDLR; SASH3; SYNGR3; TIMM23; NUP210; TUBG1; CTSL2; IGHG1; MAP4K1; ACP6; RNF141; CTSW; RNFT2; QRSL1; SIT1; PPP1R3A; TRIM14; CDCA8; HSD11B1; DENND1C; IDI1; KCNV1; ICOS; |
| **S100A7A** | SERPINB3; SERPINB4; SMR3A; ORM2; MUC4; DMBT1; GNT3; XDH; C4BPA; CEACAM1; PI3; SMR3B; SLC26A4; BTN1A1; SLC5A1; DEFB1; ORM1; |
| **S100A16** | NFAT5; DNAJC6; ZNRF1; RFK; LOC442075; TMEM170A; ZNF23; SMC2; |
| **S100B** | ANAPC5; TGFBR2; STRAP; |
| **S100G** | SYT4; CSMD3; LRTM2; VSTM2A; RAB3C; INA; CGA; CHGB; CARTPT; GPR158; SGG2; PCSK1; CHGA; EDDM3B; LOC90925; DDX25; |
| **S100P** |  |
| **S100Z** | SRD5A3; |
